# Draft Genome Sequence of the Multiple Antibiotic Resistant Pseudomonas aeruginosa PAO1-UB Subline

**DOI:** 10.1128/mra.00646-22

**Published:** 2022-08-22

**Authors:** Kah-Ooi Chua, Paul Norton, Pavlos Trus, Maria Katsikogianni, M. Julie Thornton, Kok-Gan Chan, Chien-Yi Chang

**Affiliations:** a Centre for Research in Biotechnology for Agriculture, University of Malaya, Kuala Lumpur, Malaysia; b Centre for Skin Sciences, Faculty of Life Sciences, University of Bradford, Bradford, United Kingdom; c School of Dental Sciences, Newcastle University, Newcastle upon Tyne, United Kingdom; d Polymer and Biomaterials Research Centre, School of Chemistry and Bioscience, University of Bradford, Bradford, United Kingdom; e Institute of Biological Sciences, Faculty of Science, University of Malaya, Kuala Lumpur, Malaysia; f International Genome Centre, Jiangsu University, Zhenjiang, China; University of Arizona

## Abstract

We report the draft genome sequence and antibiotic susceptibility of Pseudomonas aeruginosa strain PAO1-UB, a subline of the common reference strain PAO1. This strain was sequenced in order to provide information on the genome dynamics of PAO1 sublines and their genes conferring resistance to multiple antibiotics.

## ANNOUNCEMENT

Pseudomonas aeruginosa is a Gram-negative bacterium and an opportunistic pathogen of humans, plants, and animals which has the ability to develop resistance to multiple classes of antibiotics (1, 2). Although a high number of P. aeruginosa strains and isolates have been reported to date, PAO1 remains the most-used reference strain for Pseudomonas research (3, 4) and has been distributed and maintained in laboratories and culture collections worldwide, giving rise to different sublines. Studies have shown that different PAO1 strains individually maintained and adapted by various research groups can undergo microevolution, even if the strain originated from the same ancestral PAO strain isolated in 1955, which has been suggested to be lost (1). In many cases, strain-to-strain genomic and phenotypic variabilities have been reported (1, 5). Hereby, we performed whole-genome sequencing and antibiotic susceptibility testing of P. aeruginosa PAO1-UB, which is maintained in our laboratory collection.

The Kirby-Bauer disk diffusion assay, conducted on Oxoid nutrient broth (NB) agar at 37°C for 24 h, revealed that PAO1-UB was resistant to penicillin G, oxacillin, chloramphenicol, erythromycin, fusidic acid, novobiocin, clindamycin, and sulfamethoxazole/trimethoprim. However, PAO1-UB exhibited susceptibility to gentamycin and tetracycline (Table 1).

**TABLE 1:** Antibiotic susceptibility of Pseudomonas aeruginosa PAO1-UB using the disk diffusion method

Antibiotic	Concn on disk^*a*^	Zone of inhibition (mm)	Bacterial susceptibility
Penicillin G	1 unit	0	Resistant
Oxacillin	5 μg	0	Resistant
Chloramphenicol	25 μg	0	Resistant
Erythromycin	5 μg	0	Resistant
Gentamycin	10 μg	18	Susceptible
Tetracycline	30 μg	20	Susceptible
Fusidic acid	10 μg	0	Resistant
Novobiocin	5 μg	0	Resistant
Clindamycin	2 μg	0	Resistant
Sulfamethoxazole/trimethoprim	25 μg	0	Resistant

a The filter paper disks are about 6 mm in diameter.

Whole-genome sequencing of the strain was performed by MicrobesNG. Briefly, the genomic DNA was extracted from a 37°C overnight NB culture using solid-phase reversible immobilization (SPRI) beads (Beckman Coulter, USA). The library was prepared using the Nextera XT library prep kit (Illumina, USA) on a Hamilton Microlab STAR automated liquid handling system. The library was sequenced using an Illumina NovaSeq platform (250-bp paired-end setting). The raw data were quality filtered using Trimmomatic, and *de novo* genome assembly was performed using SPAdes v3.15.4 (6). The assembled draft genome was assessed for quality using QUAST v5.2.2 and completeness using Benchmarking Universal Single-Copy Ortholog (BUSCO) v5.3.2 (7). Genome annotation was carried out using the Prokaryotic Genome Annotation Pipeline (PGAP) (8), Prokka v1.14.6 (9), and a resistome analysis in the Comprehensive Antibiotic Resistance Database (CARD) (https://card.mcmaster.ca/) (10). Default parameters were used for all software.

The sequencing resulted in 1,312,546 quality-filtered reads (range, 36 to 251 bp). The assembled draft genome of P. aeruginosa PAO1-UB was 5,912,399 bp long and consisted of 155 contigs with 66.8% G+C content (coverage, 30×). PGAP annotation revealed 5,501 coding DNA sequences (CDS), 6 rRNAs, and 57 tRNAs. The draft genome sequence was shorter and had fewer genes than the complete genome of P. aeruginosa POA1 (GenBank accession number AE004091; 6,264,404 bp; 5,572 CDS, 13 rRNAs, and 63 tRNAs). Nonetheless, the PAO1-UB draft genome scored 99.3% completeness in the BUSCO analysis using the *Pseudomonadales* data set. Genes conferring resistance to antibiotics, such as β-lactams (*bla*_OXA-396_ [locus tag, NF546_RS12590] and a Pseudomonas-derived cephalosporinase, PDC-5 [NF546_RS26465]) and fosfomycin (*fosA*) (NF546_RS20960), were detected in the genome. The resistome analysis also identified component genes of multiple drug efflux systems that were responsible for resistance to chloramphenicol (*mexA* [NF546_RS08650], *mexB* [NF546_RS08655], and *oprM* [NF546_RS08660]) and fluoroquinolones (*mexC*-*mexD*-*oprJ* [NF546_RS17525, NF546_RS17520, NF546_RS17515]). Overall, these data on antibiotic susceptibility and from the resistome analysis of PAO1-UB will be useful for interlaboratory comparison to assess the genomic and phenotypic variability between different P. aeruginosa PAO1 sublines.

No ethical approval was required for this research.

### Data availability.

This whole-genome shotgun project has been deposited at DDBJ/ENA/GenBank under the accession number JAMWGW000000000. The version described in this paper is version JAMWGW010000000. The project data are available under the BioSample accession number SAMN29127182 and the BioProject accession number PRJNA849708. The raw sequencing reads were deposited in SRA under the accession number SRR20177986.
